# High ambient temperatures and hypertensive disorders of pregnancy: a systematic review and meta-analysis protocol

**DOI:** 10.12688/hrbopenres.14304.1

**Published:** 2026-01-19

**Authors:** Margaret M Brennan, Teresa O'Dowd, Nicholas B Brink, Danielle Travill, Cherlynn Dumbura, Carla Devantier-Du Pleiss, Ursula Gazeley, Lebohang Radebe, Jeroen de Bont, Andrew Parnell, Fergus P. McCarthy, Cathal Walsh, Noel D McCarthy, Matthew F Chersich, Darshnika Lakhoo

**Affiliations:** 1 Discipline of Public Health and Primary Care, Institute of Population Health, School of Medicine, Trinity College Dublin, Dublin, Leinster, Ireland; 2 National Health Intelligence Unit, Jervis House, Jervis Street, Health Service Executive, Dublin 1, Ireland; 3 Wits Planetary Health Research, Faculty of Health Sciences, University of the Witwatersrand Johannesburg, Johannesburg, Gauteng, South Africa; 4 National Health Improvement Team, Health Service Executive, Galway, Ireland; 5 Centre for Sexual Health and HIV/AIDS Research Zimbabwe, Harare, Harare Province, Zimbabwe; 6 Leverhulme Centre for Demographic Science, Nuffield Department of Population Health, University of Oxford, Oxford, England, UK; 7 Institute of Environmental Medicine, Karolinska Institut, Stockholm, Sweden; 8 Earth Institute and School of Computer Science, School of Mathematics and Statistics, University College Dublin, Dublin, Ireland; 9 Department of Obstetrics and Gynaecology, University College Cork, Cork, Ireland

**Keywords:** High ambient temperatures, Heat, Heatwaves, Hypertensive disorders of pregnancy, Gestational hypertension, Pre-eclampsia, Eclampsia, HELLP syndrome, Windows of susceptibility, Methodology, Climate change

## Abstract

**Background:**

Exposure to high ambient temperatures, commonly referred to as heat, has been consistently associated with adverse birth outcomes, particularly preterm birth. There is less consensus with regard to links with adverse maternal outcomes, such as hypertensive disorders of pregnancy (HDP). HDP are a leading cause of maternal mortality and morbidity, whose aetiology remains poorly understood. In the context of global warming driven by climate change, clarifying this potential association is of importance for global health.

**Objective:**

The proposed systematic review and meta-analysis aims to synthesise and critically appraise the available peer-reviewed literature on heat exposure and HDP, with specific attention paid to differences across HDP subtypes, windows of susceptibility and study methods. The findings will provide novel insights into the potential role of heat in the aetiology of HDP, and guide future research.

**Methods:**

A systematic search of PubMed, EMBASE and Web of Science will be undertaken using the detailed prespecified search strategy that was reviewed by an expert librarian. Additional texts will be identified through snowball reference searching. Two authors will independently screen titles, abstracts and full-texts, and undertake data extraction, with discrepancies resolved by a third reviewer. HDP are the primary outcomes of interest and include gestational hypertension, pre-eclampsia (de novo, superimposed, early- and late-onset), and associated complications, namely eclampsia and haemolysis, elevated liver enzymes and low platelet count (HELLP) syndrome. Secondary considerations include identifying windows of susceptibility, evaluating epidemiological designs and statistical methods employed. Findings will be summarised through narrative synthesis and quantitatively through random-effects meta-analyses where possible. Risk of bias in individual studies and confidence in the cumulative body of evidence will be assessed following the Navigation Guide Criteria. The review will be reported in accordance with the Preferred Reporting Items for Systematic Reviews and Meta-Analyses and the Meta-analyses Of Observational Studies in Epidemiology guidelines.

## Introduction

Hypertensive disorders of pregnancy (HDP), including chronic hypertension, gestational hypertension and pre-eclampsia ^1^, are estimated to affect 2–9% of pregnancies worldwide ^2, 3^, and are leading global causes of maternal mortality and morbidity ^4, 5^. Beyond the perinatal period, women with a history of HDP face elevated life-course risks of cardiovascular ^6^, renal ^7, 8^, and neurological diseases ^9, 10^, while their children experience increased mortality risks from birth to young adulthood ^11^. Despite considerable research and the identification of several risk factors, the triggers of HDP remain poorly understood, limiting the ability to accurately predict who will develop HDP ^1^. Advancing understanding of these triggers and preventing HDP is a priority, as delivery of the placenta remains the only intervention proven to initiate pre-eclampsia resolution ^3^.

Emerging evidence suggests that environmental factors such as high ambient temperatures, commonly referred to as heat, may contribute to adverse pregnancy outcomes, including HDP ^12^. A large-scale systematic review published in 2024, summarised evidence for the association between heat exposure and twenty-three maternal, fetal and neonatal outcomes including HDP ^12^. This review reported that 21 of 28 identified studies found an adverse association between heat exposure and HDP ^12^. However, because the review considered such a broad range of outcomes, HDP was assessed only as a composite outcome, leaving several important questions unanswered ^12^. These include whether heat exposure is differentially associated with HDP subtypes, the timing of exposure that may represent windows of susceptibility during pregnancy for these subtypes, and the influence that methodological differences between studies may be having. An earlier systematic review on extreme temperature and HDP likewise did not resolve these questions ^13^, and the publication of multiple new studies since underscores the need for an updated synthesis. In contrast, the evidence linking heat exposure to adverse birth outcomes, such as preterm birth, is considerably stronger ^12^.

In terms of potential mechanisms, HDP, particularly pre-eclampsia, are often regarded as placental disorders ^3^. Although mechanistic evidence from human studies is limited, heat exposure during pregnancy has been associated with various placental changes, including reduced placental weight and volume, altered expression of genes involved in inflammatory pathways and increased umbilical artery resistance indices, suggesting a plausible biological pathway through which HDP may be influenced by heat ^14– 16^ Furthermore, the association between heat exposure and cardiovascular-related mortality and morbidity is well documented ^17^, and since HDP are strongly linked with later-life cardiovascular disease ^18, 19^, heat may contribute to HDP through similar pathways.

In 2024, global temperatures exceeded 1.5°C above pre-industrial levels, marking the warmest year on record ^20, 21^. Under current greenhouse gas emission (GHGe) trajectories, projections indicate that one-third of the global population could experience mean annual temperatures exceeding 29°C by 2070, conditions currently found on only 0.8% of Earth’s land surface ^22^. In this context, understanding whether high ambient temperatures affect HDP is of crucial importance for global maternal and perinatal health. This knowledge is urgently needed to guide the prioritisation of population health interventions, including early warning systems for heatwaves and clinical guidelines for pregnancy, as well as to motivate effective climate change mitigation.

Our proposed systematic review and meta-analysis will address the gaps we have identified in the existing literature by synthesising and critically appraising the available peer-reviewed literature on heat exposure and HDP, with specific attention paid to differences across HDP subtypes, timing of exposure and study methods. This will provide novel insights into the potential role of heat in HDP aetiology and help guide priorities and approaches for future research in this field.

This systematic review aims to address three key questions. First, is exposure to high ambient temperatures associated with increased risk of hypertensive disorders of pregnancy (HDP) and related complications, including gestational hypertension, pre-eclampsia (de novo or superimposed; early- or late-onset), eclampsia and haemolysis, elevated liver enzymes and low platelets (HELLP) syndrome? In addition, does the magnitude of this association vary between HDP subtypes, and across different climate zones or country income levels, as well as by maternal factors such as age, parity, body mass index (BMI) and socioeconomic status? Second, are there windows of susceptibility during pregnancy when heat exposure increases the risk of HDP, and do these windows differ between HDP subtypes? For example, is high ambient temperatures early in pregnancy, a critical period for placentation ^3^, particularly associated with early-onset pre-eclampsia? Third, how do methodological differences between studies influence associations reported in the current evidence base?

## Methods

### Study design

This is a protocol for a systematic review and meta-analysis of observational studies which was drafted in accordance with the Preferred Reporting Items for Systematic Reviews and Meta-Analyses Protocol (PRISMA-P) guidelines ^23^. This protocol has also been registered with the International Prospective Register of Systematic Reviews (PROSPERO: CRD420251136030). If any important amendments arise, these will be documented promptly on PROSPERO. The full review will be reported in accordance with both the Preferred Reporting Items for Systematic Reviews and Meta-Analyses (PRISMA) and the Meta-analyses of Observational Studies in Epidemiology (MOOSE) checklists ^24, 25^.

### Eligibility criteria

Studies will be eligible for inclusion if they reported on the following:

- Population: Pregnant human populations.
- Exposure: Elevated or high ambient temperatures or heat (including heatwaves) or other heat indices pre-conception or during pregnancy.
- Comparator: Pregnancy population exposed to lower levels of heat
- Outcome: HDP, including gestational hypertension, pre-eclampsia (de novo or superimposed; early or late-onset), and related complications such as eclampsia and HELLP syndrome ^1^. Of note, while chronic hypertension is classified as an HDP by the International Society for the Study of Hypertension in Pregnancy (ISSHP) ^1^, it will not be included as an outcome in this systematic review, as by criteria this is present pre-conception or before 20 weeks of gestation, making it difficult to determine whether heat exposure preceded its onset. Instead we will examine whether studies have appropriately accounted for participants with chronic hypertension. This might refer to exclusion of participants with chronic hypertension, or that those with chronic hypertension are analysed separately. Outcomes may be defined using established clinical criteria, hospital records, diagnostic codes or self-reported doctor diagnosis. The method of outcome ascertainment will be documented.
- Study design: Peer-reviewed full-text articles of observational, quantitative studies (e.g. cohort, case-control, case-crossover, cross-sectional, ecological, time series)

Studies are not eligible for inclusion if:

- Population: Includes non-pregnant or non-human populations,
- Exposure: Investigates non-ambient heat exposures (e.g. hot baths or saunas) or focus solely on season without explicitly considering temperature.
- Outcome: Do not report on HDP or related complications.
- Study design: Reviews, commentaries, individual case reports, conference abstracts, qualitative studies and grey literature.
- Language: Not available in English.

### Search strategy and information sources

A comprehensive search strategy was developed using relevant Medical Subject Headings (MeSH) and free-text terms linked using Boolean operators. The strategy was reviewed by a senior librarian and is provided in Table 1. The lead author (MMB) will apply this search strategy to systematically search three large electronic databases: PubMed (National Library of Medicine), EMBASE (Elsevier) and Web of Science (Clarivate) from database inception to the date of searching, in order to identify peer-reviewed journal articles meeting inclusion criteria. In addition, key reference lists and backward and forward snowball citation searching of included articles will be conducted to identify further relevant articles.

**Table 1.** Search strategy.

("Hypertensive disorder*" OR HDP OR hypertens* OR "Chronic hypertension" OR "Gestational hypertension" OR hypertension OR “hypertension, pregnancy-induced” OR “pregnancy-induced hypertension” OR “pregnancy induced hypertension” OR "high blood pressure" OR "pre-eclampsia" OR preeclampsia OR “pre eclampsia” OR eclampsia OR "HELLP syndrome" OR HELLP OR "Haemolysis elevated liver enzymes low platelets" OR "Hemolysis elevated liver enzymes low platelets") AND
(pregnan* OR pregnant OR pregnancy) AND
(heat OR heatwave* OR “heat wave” OR "hot weather" OR "extreme heat" OR temperatur* OR "ambient temperature" OR "temperature extremes" OR "high temperature*" OR "elevated temperature" OR “hot temperature” OR “heat strain” OR “heat exposure” OR “heat stress” OR “heat index” OR “heat episode” OR “heat event”)

### Data management

Covidence, the web-based systematic review management software will be used. All references from the systematic search will be imported into Covidence. Automatic deduplication will be performed by Covidence but a reviewer will manually spot check this process for accuracy.

### Selection process

Eligibility screening will take place in two stages: title and abstract, and then full text review. At each stage, two reviewers will independently screen records for inclusion, blinded to each other’s decisions. If there are any disagreements on screening, a third reviewer will facilitate resolution of any screening disagreements via consensus discussion. All selection decisions will be recorded on Covidence, which will generate a PRISMA flow diagram that will be presented in the review.

In parallel, we will use a novel machine learning and large-language model-based method for title, abstract and full text screening ( https://github.com/drnicholasbrink/ml_review). Its results will be compared with those from the dual human reviewer process to assess the feasibility and reliability of this approach for future updates and other reviews.

### Data collection process

Data extraction will be performed in duplicate by two authors working independently. A third reviewer will assist in resolving any disagreements via consensus discussion. The data extraction form will be piloted before finalisation.

### Data items

Data will be sought for the following variables: author, year of publication, study design, study setting (e.g. country, region or city), sample size, eligibility criteria for study population, whether chronic hypertension was accounted for, availability of a study protocol and correspondence with the final analyses, exposure definition (e.g., ambient temperature, universal thermal climate index, wet bulb globe temperature, apparent temperature, other), source of exposure data (e.g., observational or reanalysis), geolocation of exposure data (e.g., linked by residential location or facility or other), spatial resolution of exposure data, exposure time windows examined (e.g. short-term [days, week], or long-term [pregnancy, trimester]), outcome definition and how ascertained, whether temporality of exposure-outcome relationship was considered and preserved, statistical approach used, reference category used in the model or unit of increase (e.g., per 1°C, or from the 75 ^th^ to 99 ^th^ percentile of temperature distribution, or other), confounders adjusted for, effect modifiers examined (if any), type of effect estimates (e.g., hazard ratio (HR), risk ratio (RR) or odds ratio (OR)), crude and adjusted effect estimates with associated confidence intervals and study limitations.

National income status will be assigned using the World Bank classification, and climate zone will be assigned based on the Köppen-Geiger climate classification according to the study setting.

### Outcomes and prioritisation

The primary outcomes are HDP, including gestational hypertension and pre-eclampsia (de novo, superimposed, early- and late-onset), and complications of HDP, such as eclampsia and HELLP syndrome ^1^. Secondary outcomes include identifying potential windows of susceptibility during pregnancy and evaluating methodological approaches used in the studies.

### Risk of bias in individual studies

Risk of bias will be assessed considering exposure assessment, outcome assessment, confounding bias, selection bias, incomplete outcome data, selective outcome reporting, conflict of interest, other sources of bias and overall risk of bias, following the Navigation Guide for systematic reviews of environmental exposures ^17, 26^.

### Data synthesis

Heterogeneity will be assessed using forest plot displaying study-specific estimates with confidence intervals, and the I² Statistic to quantify the proportion of total variability attributable to heterogeneity. Random-effects meta-analyses will be conducted for each outcome, if three or more publications of adequate quality are available. Potential sources of heterogeneity will be explored using meta-regression, provided a sufficient number of studies are available per moderator. Moderators of interest include environmental context (e.g., Köppen-Geiger climate zone, country income status), population characteristics (e.g. maternal age, parity, BMI, baseline health status, and socioeconomic status), exposure characteristics (e.g., temperature metric), windows of susceptibility assessed, and methodological features (e.g., study design, sample size, adjustment for confounders, risk of bias). A sensitivity analysis will be conducted to test the influence of each study by sequential omission. Where meta-analysis is not possible, summary statistics (e.g., median and interquartile range) will be estimated and a narrative synthesis will be undertaken, grouped by outcome, using text and tables to provide a descriptive summary of study characteristics and review findings.

### Meta-biases

Publication bias will be assessed using funnel plots, with formal statistical tests applied where appropriate (e.g., Egger's test).

### Confidence in cumulative evidence

Quality and strength of the body of evidence will be assessed across all studies using the Grading of Recommendations, Assessment, Development and Evaluation (GRADE) domains: risk of bias, indirectness of the evidence, inconsistency of findings, imprecision, publication bias, effect size, dose-response relationship and confounding, also following the Navigation Guide ^17, 26^.

### Ethics and dissemination

This review will be based on published data and does not require ethical approval. The results will be disseminated widely through presentations at academic conferences, public events, to policymakers and through publication in a peer-reviewed journal.

### Patient and public involvement

Neither patients nor other members of the public were involved in the design of this systematic review and meta-analysis. However, the lead author (MMB) will ensure communication of the results to relevant patient and public groups upon completion.

### Potential limitations

We anticipate a number of limitations that this review may face. These arise from practises within academic publishing, observational study design, ascertainment of outcome and from within the review team. First, within academic publishing, publication bias is well recognised, this may reduce the likelihood of identifying studies reporting non-significant associations, potentially influencing our findings. We will assess for its presence and discuss the potential implications. Second, study design. This research question can only be addressed in human using observational study designs due to ethical considerations. It is well established that observational studies are susceptible to issues such as residual confounding and the influence of modelling choices. Assessing how these limitations may have influenced the evidence base, and the extent to which reliable conclusions can be drawn, is a key objective of this review. Third, variation in how outcomes are defined across studies may introduce substantial heterogeneity. Lastly, language restrictions. Only studies published in English will be included due to language limitations among the review team, which may result in the exclusion of relevant studies published in other languages.

## Discussion

There is currently a lack of consensus on whether high ambient temperatures increase the risk of HDP, or whether effects differ across HDP subtypes and complications. This systematic review will exhaustively summarise and rigorously appraise the available evidence. This will provide novel insights into the potential role of elevated ambient temperatures in the aetiology of HDP, windows of susceptibility and methodological influence. Findings from this review will advance knowledge in this area and guide future targeted research in this field.

## Statements

### Data availability

There is no underlying data associated with this protocol.

PRISMA-P reporting checklist for this systematic review protocol can be accessed at Figshare: Brennan, Margaret (2025). PRISMA-P checklist for "High ambient temperatures and hypertensive disorders of pregnancy: a systematic review and meta-analysis protocol". Figshare. Dataset. https://doi.org/10.6084/m9.figshare.30850022.v1. Released under a CC BY 4.0 licence ^27^.
