# Supplementary material for: High ambient temperatures and hypertensive disorders of pregnancy: a systematic review and meta-analysis protocol
Source: HRB Open Res. 2026 Oct 6;9:12. Originally published 2026 Jan 19. [Version 2] doi: 10.12688/hrbopenres.14304.2 (PMC13669572; doi:10.12688/hrbopenres.14304.2)
Supplement: Peer review history

# Reviewer report: Reviewer response for version 2

**DOI:** 10.21956/hrbopenres.16059.r58383

**Published:** 2026-10-14

**Authors:** Huiqi Chen

**Affiliations:** 1 London School of Hygiene and Tropical Medicine, London, England, UK

Many thanks for sending the revised version. I have reviewed the revisions and am satisfied that the authors have adequately addressed my previous comments. The protocol is now sufficiently clear. I am therefore happy to update my status to **Approved**.

Is the study design appropriate for the research question?

Yes

Is the rationale for, and objectives of, the study clearly described?

Yes

Are sufficient details of the methods provided to allow replication by others?

Partly

Are the datasets clearly presented in a useable and accessible format?

Not applicable

Reviewer Expertise:

NA

I confirm that I have read this submission and believe that I have an appropriate level of expertise to confirm that it is of an acceptable scientific standard.

# Reviewer report: Reviewer response for version 1

**DOI:** 10.21956/hrbopenres.15743.r54244

**Published:** 2026-04-06

**Authors:** Claire Masters

**Affiliations:** 1 Yale University School of Public Health, New Haven, Connecticut, USA

This article presents a well-considered protocol for a systematic review and meta-analysis examining the association between high ambient temperatures and hypertensive disorders of pregnancy (HDP). HDP, which encompasses gestational hypertension, pre-eclampsia, eclampsia, and HELLP syndrome, affects around 2–9% of pregnancies worldwide. They constitute a major source of maternal (and infant) mortality and morbidity and a significant driver of healthcare costs. Despite growing evidence linking heat exposure to adverse birth outcomes such as preterm birth, the relationship between heat and HDP is less well understood.

 The authors aim to address whether heat exposure increases risk of HDP, whether certain windows of pregnancy are particularly vulnerable, and how methodological differences across studies affect reported findings.

 While the protocol includes a broad range of heat-related search terms, it does not define minimum thresholds or cut points for what constitutes high ambient temperature across different contexts. I feel this is necessary. Without at least a working definition; for example, a minimum absolute temperature threshold (e.g., >25°C or >30°C) or a percentile-based approach (e.g., above the 90th percentile of local temperature distribution), it is unclear how studies will be compared or pooled in meta-analysis. The authors should specify, even cautiously, what exposure threshold will be considered the minimum for inclusion, or explicitly justify why no threshold is applied.

 Similarly, the protocol does not address exposure duration. Heat exposure over a single day may have very different biological implications than sustained exposure over a week or trimester. The authors should specify whether there is a minimum duration of exposure required for inclusion, and how duration will be captured and reported across studies. This is particularly relevant given their stated interest in windows of susceptibility, where duration and timing likely interact.

 Overall, this is a timely and well-conceived protocol addressing an important gap in the literature, and its findings are eagerly anticipated.

Is the study design appropriate for the research question?

Yes

Is the rationale for, and objectives of, the study clearly described?

Yes

Are sufficient details of the methods provided to allow replication by others?

No

Are the datasets clearly presented in a useable and accessible format?

Yes

Reviewer Expertise:

Envrionmental epidemiology; perinatal environmental exposure research, heat and pregnancy ouctomes, demography and fertility

I confirm that I have read this submission and believe that I have an appropriate level of expertise to confirm that it is of an acceptable scientific standard.

# Response

**Published:** 2026-09-25

**Authors:** Margaret Brennan

**Affiliations:** Public Health and Primary Care, Trinity College Dublin School of Medicine, Dublin, Leinster, Ireland

**RESPONSE TO REVIEWER 2**

*2.1. This article presents a well-considered protocol for a systematic review and meta-analysis examining the association between high ambient temperatures and hypertensive disorders of pregnancy (HDP). HDP, which encompasses gestational hypertension, pre-eclampsia, eclampsia, and HELLP syndrome, affects around 2–9% of pregnancies worldwide. They constitute a major source of maternal (and infant) mortality and morbidity and a significant driver of healthcare costs. Despite growing evidence linking heat exposure to adverse birth outcomes such as preterm birth, the relationship between heat and HDP is less well understood. The authors aim to address whether heat exposure increases risk of HDP, whether certain windows of pregnancy are particularly vulnerable, and how methodological differences across studies affect reported findings.*

**Author response: **Thanks for the kind words and for taking the time to review our protocol. We have sought to address the useful points you raised below and believe that the changes have substantially improved the protocol.

*2.2. While the protocol includes a broad range of heat-related search terms, it does not define minimum thresholds or cut points for what constitutes high ambient temperature across different contexts. I feel this is necessary. Without at least a working definition; for example, a minimum absolute temperature threshold (e.g., >25°C or >30°C) or a percentile-based approach (e.g., above the 90th percentile of local temperature distribution), it is unclear how studies will be compared or pooled in meta-analysis. The authors should specify, even cautiously, what exposure threshold will be considered the minimum for inclusion, or explicitly justify why no threshold is applied.*

**Author response: **Thanks for this feedback. Eligibility for the review is not restricted to a specific exposure threshold – any study reporting on the association between heat exposure and HDP will be eligible for inclusion in the review to ensure we provide a comprehensive overview of the existing evidence base. All studies will be summarised narratively.

 However, for meta-analytic pooling, we will apply a percentile-based definition: high ambient temperatures will be defined as ≥95th percentile of the local temperature distribution. This ensures estimates pooled in meta-analyses are comparable and that temperature exposure is contextualised relative to the local temperature distribution, rather than relying on absolute values that may correspond to different levels of physiological stress across diverse climatic settings. Alternative percentile thresholds (e.g. ≥90th and ≥99th percentile) will be evaluated in sensitivity analyses where study numbers permit.

*2.3. Similarly, the protocol does not address exposure duration. Heat exposure over a single day may have very different biological implications than sustained exposure over a week or trimester. The authors should specify whether there is a minimum duration of exposure required for inclusion, and how duration will be captured and reported across studies. This is particularly relevant given their stated interest in windows of susceptibility, where duration and timing likely interact.*

**Author response: **Thanks for this feedback. We agree that exposure duration is likely biologically important. No minimum duration of exposure was specified for inclusion; the exposure duration used in each study (e.g. weekly, four-weekly, trimester) was recorded during data extraction for each study and will be reported in a supplementary table with the review. Given the anticipated number of studies, we do not expect to have sufficient data to formally test duration of exposure as a moderator of effect size in meta-analyses. This will be reported as a limitation of the review, alongside narrative discussion of how duration may interact with the gestational timing of exposure.

*2.4. Overall, this is a timely and well-conceived protocol addressing an important gap in the literature, and its findings are eagerly anticipated.*

**Author response: **Thanks very much again for your time and kind words!

# Reviewer report: Reviewer response for version 1

**DOI:** 10.21956/hrbopenres.15743.r54113

**Published:** 2026-03-27

**Authors:** Huiqi Chen

**Affiliations:** 1 London School of Hygiene and Tropical Medicine, London, England, UK

This manuscript presents a protocol for a systematic review and meta-analysis examining the association between high ambient temperatures and hypertensive disorders of pregnancy (HDP). The topic is timely given the warming climate and the significant burden of HDP on maternal and perinatal health. Overall the protocol is well-structured and the methods are largely sound. Several points warrant clarification.

 Major comments:

 1. Exposure metric: The protocol lists multiple heat exposure metrics (ambient temperature, UTCI, WBGT, etc.) but does not specify how studies using different metrics will be handled in the meta-analysis. For example, specify whether the authors intend to standardise effect estimates before pooling. Also, some studies may report non-linear temperature–health relationships, which raises the question of how effect estimates will be extracted for meta-analysis.

 2. The protocol aims to investigate windows of susceptibility during pregnancy; however, clarification is needed on how heterogeneous exposure windows will be handled both within and across studies.

 - Within-study (multiple windows):

 Many studies report estimates for multiple exposure windows (e.g., different trimesters or lag structures). The authors should pre-specify how these will be handled during data extraction and meta-analysis (e.g., whether all windows will be extracted and analysed separately, or whether a single estimate will be selected based on predefined criteria). 

 - Between-study (inconsistent window definitions):

 Studies may define exposure windows at different temporal scales (e.g., daily exposure, trimester averages, or whole-pregnancy exposure). I suggest clarifying whether analyses will be stratified by exposure window definitions and whether separate meta-analyses will be conducted for different gestational periods. In addition, it would be helpful to describe how overlapping or non-comparable exposure windows across studies will be managed.

 3. Effect measure: Studies in this area report a variety of effect measures (OR, RR, HR) with heterogeneous reference categories (per 1°C increase, per IQR, heatwave vs. non-HW). The protocol should explicitly state whether conversion between effect measure types will be attempted, on what assumptions, and whether analyses will be stratified accordingly.

 4. Non-independence from multi-city/multi-country studies

 Large epidemiological studies in this domain frequently contribute data from multiple cities or countries, raising the issue of statistical non-independence when multiple estimates from the same study are pooled. The protocol does not address this, which needs to be specified.

 Minor comments:

 1. The eligibility criteria exclude chronic hypertension as an outcome but do not address how studies that fail to report whether participants with pre-existing hypertension were excluded will be handled. 

 2. The protocol indicates that extracted data will include confounders adjusted for in the original studies, but it is not clear how this information will be incorporated into the synthesis.

 3. The use of an LLM-based screening tool is a methodologically interesting addition. Please clarify how the ML screening results will be evaluated, whether they will influence inclusion decisions, and whether the ML component will be reported separately as a methodological study.

 4. The threshold for "sufficient number of studies" to conduct meta-regression is not defined.

Is the study design appropriate for the research question?

Yes

Is the rationale for, and objectives of, the study clearly described?

Yes

Are sufficient details of the methods provided to allow replication by others?

Partly

Are the datasets clearly presented in a useable and accessible format?

Not applicable

Reviewer Expertise:

Environmental epidemiology, Climate change and health, Maternal and child health

I confirm that I have read this submission and believe that I have an appropriate level of expertise to confirm that it is of an acceptable scientific standard, however I have significant reservations, as outlined above.

# Response

**Published:** 2026-09-25

**Authors:** Margaret Brennan

**Affiliations:** Public Health and Primary Care, Trinity College Dublin School of Medicine, Dublin, Leinster, Ireland

**RESPONSE TO REVIEWER 1**

 1.1. *This manuscript presents a protocol for a systematic review and meta-analysis examining the association between high ambient temperatures and hypertensive disorders of pregnancy (HDP). The topic is timely given the warming climate and the significant burden of HDP on maternal and perinatal health. Overall the protocol is well-structured and the methods are largely sound. Several points warrant clarification*

**Author response:** Thank you for your kind words and for raising these points for clarification. We have sought to comprehensively address the points you raised and believe this has strengthened the protocol.

 1.2. *Exposure metric: The protocol lists multiple heat exposure metrics (ambient temperature, UTCI, WBGT, etc.) but does not specify how studies using different metrics will be handled in the meta-analysis. For example, specify whether the authors intend to standardise effect estimates before pooling. Also, some studies may report non-linear temperature–health relationships, which raises the question of how effect estimates will be extracted for meta-analysis.*

**Author response: **Thank you for raising these important points which we have addressed below and updated the protocol accordingly.

 First regarding temperature metrics: as strong correlation and similar predictive ability for mortality has been demonstrated between temperature metrics (Barnett AG et al, Environ Res, 2010), different temperature metrics (e.g. mean, minimum, maximum ambient temperature, universal thermal climate index (UTCI), heat index, wet bulb globe temperature (WBGT), physiological equivalent temperature (PET) etc.,) will be treated as comparable in meta-analyses. This approach is consistent with recent high-impact systematic reviews examining the health impacts of high ambient temperature (Lakhoo DP et al, Nat Med, 2025 and Liu J, Lancet Planet Heal, 2022). The potential influence of metric heterogeneity will be examined in sensitivity analyses, with metric-specific analyses conducted where study numbers permit.

 Second regarding standardisation of effect estimates: studies will be eligible for meta-analysis if they report adjusted odds ratios (ORs), hazard ratios (HR) or risk ratios (RR) with associated confidence intervals. Given the anticipated low prevalence of the outcomes of interest, ORs, HRs and RRs are considered reasonable approximations of the relative risk, consistent with the rare disease assumption. Accordingly, all effect estimates will be interpreted as measures of relative risk and pooled as such in meta-analyses. This approach is consistent with a recent high-impact systematic review (Lakhoo DP et al, Nat Med, 2025). The potential influence of effect estimate heterogeneity will be examined in sensitivity analyses.

 Third, regarding extracting effect estimates for pooling in meta-analysis: effect estimates will be sought for high versus lower temperature exposure, defined using percentile thresholds. This approach ensures that temperature exposure is contextualised relative to the local temperature distribution, rather than relying on absolute values that may correspond to different levels of physiological stress across diverse climatic settings. In addition, percentile-based definitions enhance comparability across studies using different temperature metrics.

 A ≥95 ^th^ percentile threshold will be used to represent high temperature, as this is the most reported threshold in the literature. Alternative thresholds (e.g. ≥90 ^th^ and ≥99 ^th^ percentile) will be examined in sensitivity analyses, where sufficient studies are available. The reference category used for comparison (“lower” temperature) will be extracted as defined in each original study (e.g. median temperatures or all temperatures below the high-temperature threshold), given the heterogenous approaches taken in the literature. This heterogeneity will be documented and its potential influence explored in sensitivity analyses.

 Where studies report continuous exposure-response relationships (linear or non-linear), effect estimates comparing percentile thresholds will be extracted when available; where this is not already reported in the paper, authors will be contacted to provide estimates at a comparable exposure contrast. Studies from which a compatible estimate cannot be obtained will be excluded from meta-analyses but described narratively.

 All studies reporting on the association between heat, however defined, on HDP will be described narratively to ensure this review provides a comprehensive synthesis of the existing evidence base.

 ​​​​​​​1.3. *The protocol aims to investigate windows of susceptibility during pregnancy; however, clarification is needed on how heterogeneous exposure windows will be handled both within and across studies.* *Within-study (multiple windows):*

*Many studies report estimates for multiple exposure windows (e.g., different trimesters or lag structures). The authors should pre-specify how these will be handled during data extraction and meta-analysis (e.g., whether all windows will be extracted and analysed separately, or whether a single estimate will be selected based on predefined criteria).*

*Between-study (inconsistent window definitions):*

*Studies may define exposure windows at different temporal scales (e.g., daily exposure, trimester averages, or whole-pregnancy exposure). I suggest clarifying whether analyses will be stratified by exposure window definitions and whether separate meta-analyses will be conducted for different gestational periods. In addition, it would be helpful to describe how overlapping or non-comparable exposure windows across studies will be managed.*

**Author response: **Thank you for raising these important questions.

 Of note, at this point, data extraction is complete. Effect estimates have been extracted for all exposure time windows reported in each primary study. Narrative synthesis and meta-analyses are pending.

 For the review synthesis, we will examine short- and long-term heat exposure windows separately.

 In accordance with Lakhoo et al, short-term exposure is defined as heat exposure occurring within approximately one month, with two subcategories:

- Preceding admission with the outcome or diagnosis of the outcome
- Delivery with the outcome.

Long-term heat exposure is defined as from pre-pregnancy through to gestational week 19. This captures the critical window for placentation, which we hypothesise may be most relevant for heat exposure. Furthermore, gestational hypertension and pre-eclampsia by definition arise from 20 weeks’ gestation onward, therefore examining exposure up to gestational week 19 ensures preservation of exposure-outcome temporality. To examine potential windows of susceptibility, the following long-term exposure windows will be examined in this review.

- Pre-pregnancy
- Gestational weeks 0-3
- Gestational weeks 4-7
- Gestational weeks 8-11
- Gestational weeks 12-15
- Gestational weeks 16-19

Where a primary study reports multiple relevant estimates (e.g. weekly effect estimates) for the review exposure windows, the midpoint estimate will be selected, ensuring each study contributes only one estimate per meta-analysis. A supplementary table will be provided with the published review describing this mapping transparently.

 ​​​​1.4. *Effect measure: Studies in this area report a variety of effect measures (OR, RR, HR) with heterogeneous reference categories (per 1°C increase, per IQR, heatwave vs. non-HW). The protocol should explicitly state whether conversion between effect measure types will be attempted, on what assumptions, and whether analyses will be stratified accordingly*.

**Author response: **Thank you for these questions. These are addressed in our response to comment 1.2.

*1.5. Non-independence from multi-city/multi-country studies. Large epidemiological studies in this domain frequently contribute data from multiple cities or countries, raising the issue of statistical non-independence when multiple estimates from the same study are pooled. The protocol does not address this, which needs to be specified.*

**Author response: **Thank you for raising this point. Where a study reports a single overall estimate combining multiple cities or countries, this pooled estimate will be extracted for inclusion in the main meta-analysis. Where a study reports only city- or country-specific estimates without an overall pooled estimate, random-effects meta-analysis will be used to derive study-level estimates, which will then be included in review meta-analyses. This is now specified in the revised protocol.

 ​​​​​​​1.6. *The eligibility criteria exclude chronic hypertension as an outcome but do not address how studies that fail to report whether participants with pre-existing hypertension were excluded will be handled.*

**Author response: **Thanks for raising this point. We will examine the potential influence of pre-existing hypertension by conducting meta-analyses with and without studies that failed to report whether participants with pre-existing hypertension were excluded, where study numbers permit. This is now specified in the revised protocol.

 ​​​​​​​1.7. *The protocol indicates that extracted data will include confounders adjusted for in the original studies, but it is not clear how this information will be incorporated into the synthesis.*

**Author response: **Thanks for this feedback. We extracted confounders adjusted for in accordance with recommendations from the Cochrane Handbook (Chapter 6, section 6.3) which advises that adjusted estimates are preferable as these usually reduce the impact of confounding and that the variables used for adjustment should be recorded. The most stringently controlled effect estimates (i.e. those from the model adjusting for more factors) will be preferentially included in our meta-analyses in accordance with approaches adopted in recent high-impact systematic reviews. (D’Antonio F et al, The Lancet Obstetrics, Gynaecology & Women’s Health, 2026). This is now specified in the revised protocol. Of note, information on confounding also informs the risk of bias and confidence assessments.

 ​​​​​​​1.8. *The use of an LLM-based screening tool is a methodologically interesting addition. Please clarify how the ML screening results will be evaluated, whether they will influence inclusion decisions, and whether the ML component will be reported separately as a methodological study.*

**Author response: **Thanks for this query. The machine learning (ML) and large-language model (LLM) screening results will be compared against studies obtained via dual human screening which remains the gold standard. ML/LLM screening results will not directly determine study inclusion, final inclusion decisions will be made by human reviewers. However, any studies uniquely identified by the ML/LLM approach will be subject to additional human review, ensuring potentially relevant studies are not overlooked. We will report the sensitivity and specificity of the ML/LLM approach, as well as comparing the time taken to complete screening relative to dual human screening. This ML/LLM methodology is currently being developed by one of the co-authors, Dr Nicholas B. Brink, and will be fully reported as a separate methodological study, currently in preparation.

 This is now specified in the protocol.

 ​​​​​​​1.9. *The threshold for "sufficient number of studies" to conduct meta-regression is not defined.*

**​​​​​​​Author response:** Thanks for this feedback. As per the Cochrane Handbook, meta-regression should only be conducted if there are more than ten studies in a meta-analysis. This is now specified in the protocol.
